# Natural Killer Cells in Multiple Sclerosis: Entering the Stage

**DOI:** 10.3389/fimmu.2022.869447

**Published:** 2022-04-06

**Authors:** Jarne Beliën, An Goris, Patrick Matthys

**Affiliations:** ^1^Department of Neurosciences, Laboratory for Neuroimmunology, Leuven Brain Institute, KU Leuven, Leuven, Belgium; ^2^Department of Microbiology, Immunology and Transplantation, Laboratory of Immunobiology, Rega Institute for Medical Research, KU Leuven, Leuven, Belgium

**Keywords:** multiple sclerosis, natural killer (NK) cells, autoimmune disorders, experimental autoimmune encephalomyelitis (EAE), immune cell heterogeneity, multiple sclerosis genetics

## Abstract

Studies investigating the immunopathology of multiple sclerosis (MS) have largely focused on adaptive T and B lymphocytes. However, in recent years there has been an increased interest in the contribution of innate immune cells, amongst which the natural killer (NK) cells. Apart from their canonical role of controlling viral infections, cell stress and malignancies, NK cells are increasingly being recognized for their modulating effect on the adaptive immune system, both in health and autoimmune disease. From different lines of research there is now evidence that NK cells contribute to MS immunopathology. In this review, we provide an overview of studies that have investigated the role of NK cells in the pathogenesis of MS by use of the experimental autoimmune encephalomyelitis (EAE) animal model, MS genetics or through *ex vivo* and *in vitro* work into the immunology of MS patients. With the advent of modern hypothesis-free technologies such as single-cell transcriptomics, we are exposing an unexpected NK cell heterogeneity, increasingly blurring the boundaries between adaptive and innate immunity. We conclude that unravelling this heterogeneity, as well as the mechanistic link between innate and adaptive immune cell functions will lay the foundation for the use of NK cells as prognostic tools and therapeutic targets in MS and a myriad of other currently uncurable autoimmune disorders.

## Introduction

Multiple sclerosis (MS) is a chronic autoimmune disorder of the central nervous system (CNS), primarily affecting young adults (1). In this age group, it forms the most common cause of non-traumatic disability (2). The first stages of the disease are characterized by transient inflammation of the brain and spinal cord which damages the myelin and axons, resulting in episodes of neurological dysfunction. As a result of the relatively young age at onset, the lifelong accumulation of disability, the increasing worldwide prevalence and the lack of a curative treatment, MS continues to pose a major socioeconomic burden (3, 4). Current disease-modifying therapies (DMTs) in MS and autoimmune disorders in general are targeting primarily the adaptive immune system, i.e. T and B cells (5–7). However, the contribution of innate immune cells such as natural killer (NK) cells is now gaining increasing attention in a myriad of autoimmune disorders (8–12), including MS (13–16).

NK cells are large granular lymphocytes best known for their capacity to kill virally-infected, malignant and stressed cells (17). They are generally divided into two main subsets, based on the surface expression of CD56 and FcγRIII (CD16): the cytotoxic CD56^dim^CD16^+^ (CD56^dim^) and the regulatory CD56^bright^CD16^-^ (CD56^bright^) NK cells (18). In peripheral blood (PB), the CD56^dim^ NK cells account for up to 90% of all NK cells. They are considered the more mature effector subset with variable expression of maturation markers such as CD57 and KLRG1 and high cytotoxic activity mainly through their production of perforin and granzymes A and B (19–21). This subset fulfills the canonical NK cell role of immune surveillance: scanning cells for infection, stress or malignancy. The expression of CD16 allows this subset to engage in antibody dependent cellular cytotoxicity (ADCC) (17). The CD56^bright^ NK cells in contrast constitute the majority of NK cells in secondary lymphoid organs and inflammatory sites (22, 23). These cells are considered less mature and generally more immunoregulatory in nature in part by virtue of their greater cytokine production, most importantly interferon (IFN)-γ (22). The distinct functionalities and distribution of NK cell subsets, both in health and in autoimmune disease, are the result of distinct repertoires of both activating and inhibitory NK cell receptors as well as chemokine receptors (18, 24, 25). For example, whereas CD56^dim^ NK cells express CXCR1, CXCR2, CX3CR1 and S1P5, CD56^bright^ NK cells express CCR7 and CXCR3. In addition, CD56^bright^ NK cells show higher expression of inhibitory major histocompatibility complex (MHC) class 1-binding receptor NKG2A as well as activating receptor NKp46, while CD56^dim^ NK cells are mainly characterized by higher levels of killer immunoglobulin-like receptors (KIR). Activation of NK cells is mediated by a balance of signals received through their inhibitory and activating receptors which are triggered by various ligands present on potential target cells (19). Interestingly, an expanding amount of literature is now describing various NK cell subsets portraying adaptive-like immunological memory and long-lived antigen specificity (26, 27). Three main types of such adaptive NK cells have been described to date: (i) NK cells found in the murine liver displaying long-lived memory to haptens and viral antigens; (ii) human NKG2C^+^ and murine Ly49H^+^ NK cells displaying cytomegalovirus (CMV) antigen-specificity and enhanced recall responses; (iii) human and murine cytokine-activated NK cells showing memory-like “trained” features such as enhanced cytotoxicity and production of IFN-γ upon restimulation (28). A more detailed description of all NK cell subtypes and their distinct characteristics falls outside the scope of this review and can be consulted elsewhere (19).

**Figure 1:**
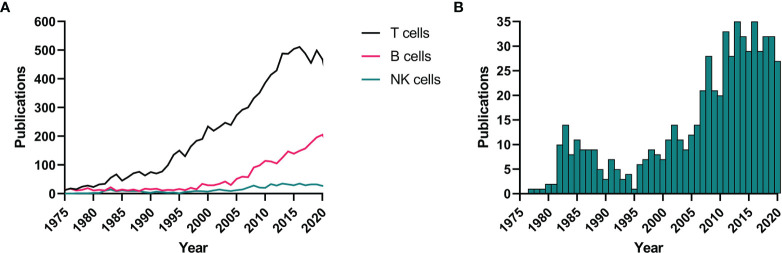
NK cells in MS literature. A systematic literature search in PubMed (NCBI) was performed using the terms “T cells”, “B cells”, or “natural killer cells” + “multiple sclerosis”. Results are shown for all three cell types from 1975 to 2020 **(A)** as well as a close-up for the NK cell results **(B)**. NK, natural killer; MS, multiple sclerosis.

Up until the last decade, NK cells have largely been ignored in the MS field and remain as of today in the shadows of the more studied adaptive T and B cells (**Figure 1A**). Apart from some exploratory studies in the 1980s (29–34), literature on the contribution of NK cells to MS immunopathogenesis is only recently starting to accumulate (**Figure 1B**). New methodologies and state-of-the art technologies have allowed for clear evidence being found in different lines of research. In this review, we provide a non-exhaustive overview of such findings from animal models, genetics, *ex vivo* and *in vitro* work. We point out the implications of these findings for the use of NK cells as prognostic tools and therapeutic targets in MS and we summarize what work remains and highlight the most pressing open questions that need answering before we can hope to include NK cells in standard clinical practice.

## A Regulatory Role for NK Cells in Animal Models of MS

A commonly studied animal model is experimental autoimmune encephalomyelitis (EAE), in which susceptible rodents develop CNS autoimmunity upon immunization with myelin-derived self-antigens emulsified in complete Freund’s adjuvant (CFA) (35). The majority of EAE studies have reported a regulatory role for NK cells. The mechanisms attributed to such regulatory action of NK cells in EAE include general T cell suppression (36–38), killing of autologous autoreactive T cells (39, 40), inhibition of the differentiation of myelin-reactive T helper type 1 (Th1) or 17 (Th17) cells in the CNS (41, 42) and secretion of neurotrophic factors (43).

In contrast to these studies, some work has been more suggestive of a detrimental role for NK cells. Researchers have postulated that the presence of NK cells, and more specifically NK cell-derived IFN-γ, is a prerequisite for development of EAE pathology (44–46). However, this concept is in strong contrast with the numerous findings that mice in which IFN-γ is depleted (using neutralizing antibodies or through genetic modification) exhibit an exacerbated form of EAE or develop the disease in strains that are normally not susceptible to develop EAE, such as the BALB/c strain (47, 48).

The contradictory reports on the role of NK cells in EAE may be attributed to the use of different animal backgrounds, divergent immunization procedures or NK cell depletion performed at different time points during development of the disease as reported by Winkler-Pickett et al. (46). Moreover, an explanation for these discrepancies might be found in the long-standing underappreciation of NK cell heterogeneity, as has been suggested by Gandhi et al. already in 2010 (49). Yet today, the independent and discrete functions of different NK cell subsets in different physiological compartments remain largely ignored in animal studies (50).

## MS Genetics Alludes a Role for NK Cells

In the last decade, the field has seen an expansion in the knowledge on the genetic basis of MS, mainly by virtue of the collaborative efforts of the International Multiple Sclerosis Genetics Consortium (IMSGC) (51–54). The most recent genomic map comprises 200 common genetic risk factors for MS on autosomes, the first one on the X-chromosome and 32 independent effects in the MHC region (55). For the first time, a systematic analysis of these risk factors showed significant enrichment for MS susceptibility loci not only in genes relating to cells of the adaptive immune system, but also in those relating to cells of the innate immune system, including NK cells. In line with this finding, several individual risk genes that appear associated with NK cell activity have been identified in MS. An example is *CD226* (55–59) which encodes for DNAX accessory molecule (DNAM)-1, a cell surface glycoprotein expressed on cytotoxic lymphocytes such as NK cells and CD8^+^ T cells, mediating their activation and killing of target cells (60, 61). The risk haplotype is associated with lower CD226 cell surface expression and MS patients have shown lower CD226 levels than healthy controls (59, 62). Interestingly, CD226 is known to be crucial for the NK cell-mediated elimination of activated T cells (63).

A genome-wide screen for low-frequency risk variants identified three novel associations with MS, including rs35947132 (p.Ala91Val) in the *PRF1* gene (54). This variant was previously reported in an Italian candidate-gene study (64), and has been confirmed in an independent cohort in Sardinia (65). *PRF1* encodes perforin, a key component of the cytotoxic pathway used by, although not exclusively, NK cells (54, 66). The p.Ala91Val variant is thought to predispose to late-onset familial hemophagocytic lymphohistiocytosis type 2 (FHLH2), a disease resulting from genetic cytotoxic defects in NK cells and CD8^+^ T cells and involving a massive cytokine storm, including release of IFN-γ (67, 68). Approximately 10% of Europeans carry this variant, which results in protein misfolding, reduced stability and, consequently, partial loss of perforin lytic activity (69). In healthy individuals, it is associated with a 35% reduction in NK cell cytotoxicity, increased overall lymphocyte count and specific augmentation of the cytotoxic memory T-cell compartment (65, 70).

Large-scale genetic studies have shown that genetic risk for MS is dominated by a series of MHC class II risk alleles, while protective effects have been attributed to a number of MHC class I alleles (53). The underlying mechanisms of action of these findings remain elusive. Apart from the classically considered T-cell receptors on CD8^+^ T cells, NK cells also express receptors that enable them to respond to MHC class I molecules, with the most important ones being the KIRs, C-type lectin-like CD94/NKG2 family and the leukocyte immunoglobulin-like receptors (LILR), such interactions have been put forward as a possible pathway for NK cell involvement in CNS disorders (24, 71). These highly polymorphic genetic regions, including structural variation, are not well captured by genotyping arrays but powerful bioinformatic tools such as large-scale imputation methods (72) and new technologies such as long-read sequencing platforms (73) now enable interrogating their contribution to MS.

## Immunoregulatory CD56^bright^ NK Cells in MS Patients

In accordance with findings from animal models and genetics, studies analyzing the cellular composition of PB and cerebrospinal fluid (CSF) in patients and healthy controls mostly point towards an immunoregulatory role for NK cells in MS. Several studies have revealed the presence of NK cells in the CSF and CNS plaques of MS patients (74–79). One of these observed NK cells expressing granzyme K, which is mainly expressed by the CD56^bright^ NK cells (80), in active MS lesions in close proximity to T cells (81). Two studies (81, 82), together investigating 152 MS patients and 42 non-inflammatory controls, described a significant increase of CD56^bright^ NK cells and, to a lower extent, CD56^dim^ NK cells in the CSF of MS patients. This was recently confirmed in periventricular tissue of MS patients *versus* controls and in periventricular lesions *versus* normal appearing white matter (83). The first study also reported a significant decrease of peripheral NK cells, predominantly the CD56^dim^ subset (81). As therapeutic modulation of IL-2 receptor signaling in these patients significantly increased the peripheral CD56^bright^ subset but not the CD56^dim^ subset, the authors speculate that NK cell maturation is impaired in MS and NK cells are stuck in the more immature CD56^bright^ stage (81). Even though a deeper mechanical understanding gained by further functional studies is certainly needed, such efforts have put NK cells, specifically the CD56^bright^ subset, on the map of MS immunopathology.

## Appreciating NK Cell Heterogeneity in MS

**Figure 2:**
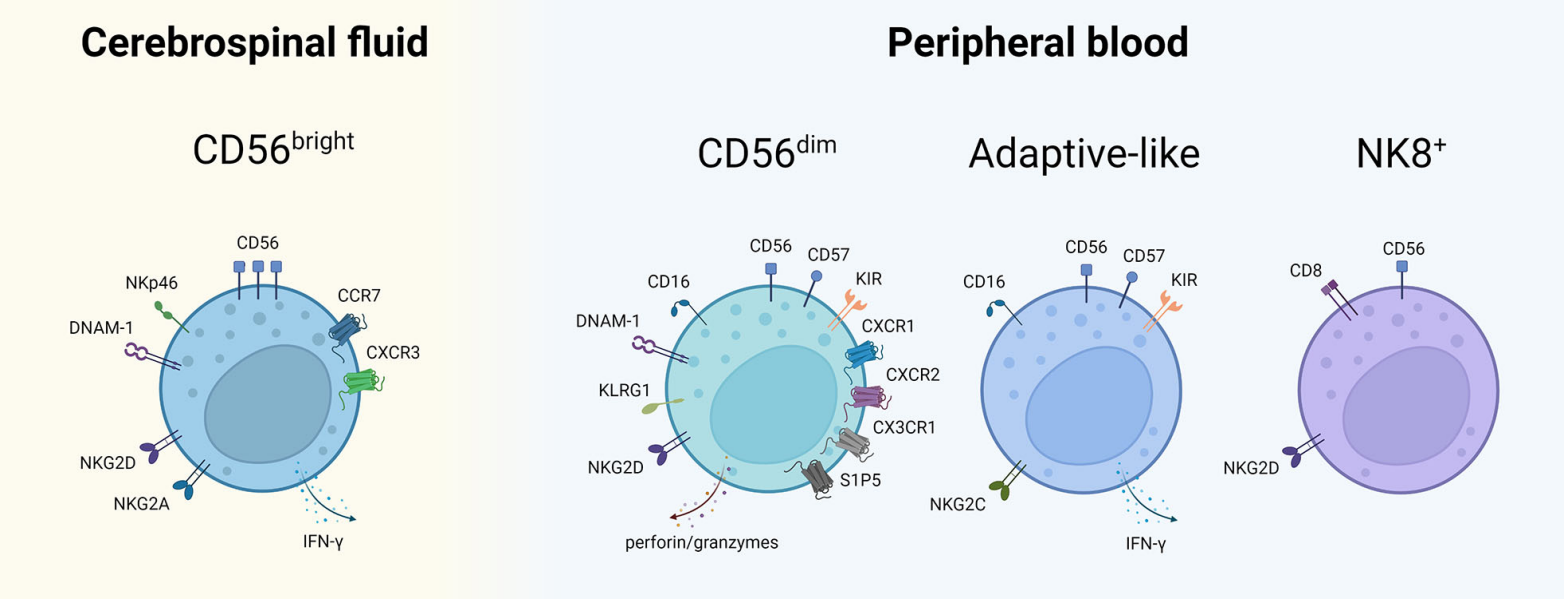
NK cell subsets described in CSF and PB of MS patients. NK cell subsets with their characteristic cell surface markers described thus far in MS. The subsets are placed in the compartment where their putative role in MS has predominantly been described. Of note: the mentioned markers are non-exhaustive, the compartmentalization is not absolute and mutual exchange between CSF and PB is possible. NK, natural killer; CSF, cerebrospinal fluid; PB, peripheral blood; NKT, natural killer T; CD, cluster of differentiation; DNAM-1, DNAX accessory molecule 1; IFN, interferon; CCR, CC-chemokine receptor; CXC, chemokine receptor; KLRG1, killer cell lectin-like receptor G1; KIR, killer immunoglobulin-like receptor; S1P5, sphingosine-1-phosphate receptor 5; NK8^+^, CD8^+^ natural killer cell. Created with BioRender.com.

Apart from the classical CD56^bright^ and CD56^dim^ NK cells, more recent work has identified several other distinct NK cells subsets in MS (**Figure 2**). De Jager et al. described an NK cell subset, defined as CD8^dim^CD56^+^CD3^-^CD4^-^, which was found to be reduced in PB of untreated MS patients as well as patients with clinically isolated syndrome (CIS) (84). More recently, McKinney et al. also described a population of ‘NK8^+^ cells’ (CD3^−^CD56^+^CD8^+^) in PB associated with a more favorable clinical outcome in MS (85). Whether or not these two studies are referencing the same NK cell subset is unclear. In another study, a CMV-driven expansion of adaptive-like NKG2C^+^ NK cells in PB was correlated with a decreased risk of disability progression (86). Such findings are starting to blur the lines between adaptive and innate immune subsets and stress the need for a better appreciation of immune cell plasticity and heterogeneity.

Recent technological advancements in the field of single-cell transcriptomics will be pivotal therein as they exploit hypothesis-free transcriptomic profiles instead of predefined cell marker subsets (87, 88). Such technologies have been able to distinguish up to 7 distinct NK cell subsets, questioning the classical two-subset (CD56^bright/dim^) view (89–91). However, the available single-cell RNA sequencing (scRNA-seq) studies in MS remain scarce and due to the low number of samples and low resolution, these studies often still limit their analyses of the NK cells to the two main subsets (92–94). In general, scRNA-seq studies confirm the lower abundance of total NK cells in CSF *versus* PB, with a cluster most likely corresponding to CD56^bright^ NK cells being the predominant NK cell subset in CSF. Schafflick et al. (92) observed an increase in the cluster abundance of two NK cell subsets, most likely corresponding to CD56^bright^ and CD56^dim^ NK cells, in CSF of MS patients (n = 4) as compared to controls (n = 4), in line with previous cytometric analyses described above (81, 82). Ramesh et al. were unable to observe a significant change in total NK cells in the CSF of MS patients (n = 10) as compared to controls (n = 3) and had no further resolution of NK cell subsets (94). More generally, these first studies aptly illustrate the importance of going beyond the canonical immune cell populations and recognizing the potential of formerly unidentified/underappreciated subsets as critical players in MS immunopathology.

## More Mechanistic Work Is Needed

The proposed mechanisms by which the aforementioned NK cell subsets exert their immunoregulatory function in MS are manifold. Several *in vitro* studies have shown that upon activation, T cells upregulate NKG2D ligands, thus becoming more susceptible to NK cell-mediated autologous killing (95–98). In MS, Jiang and colleagues have described a pivotal role for granzyme K in the immunomodulation by CD56^bright^ NK cells (99). These authors showed that eliminating granzyme K profoundly inhibited the ability of CD56^bright^ NK cells to lyse activated syngeneic T cells, thus failing to control autoimmunity. In addition, NK cells have been shown to kill other MS-relevant cell types, such as microglial cells by use of the NKG2D as well as the NKp46 receptor pathways (100), autologous immature myeloid cells *via* NKp30, NKp46 and DNAM-1 (101–103) and activated macrophages through NKG2D as well (104). Lastly, apart from direct cell-mediated cytotoxicity, NK cells are known to modulate adaptive immunity by cytokine production (105). Evidently, more functional assays will be needed to determine which of these mechanisms is used by exactly which NK cell subset to modulate adaptive immunity and control autoimmunity in MS.

## NK Cells in the MS Clinic

Several MS treatments have been described to affect NK cell populations. A systematic overview of the known effects of currently approved DMTs for MS on the NK cell repertoire is beyond the scope of this review and the authors refer to a recent review of Laroni and Uccelli (16). From a historical perspective, the effects of daclizumab, an anti-CD25 mAb that blocks the high-affinity IL-2 receptor on activated T cells, have been particularly interesting. Bielekova et al. showed that the beneficial effects of daclizumab could in part be attributed to an expansion of the CD56^bright^ NK cells, as well as an enhancement of their cytotoxic potential and regulatory capacity (106, 107). The concomitant killing of T cells is mediated by the mechanisms described in previous sections such as increased expression of granzyme K in NK cells and reversal of the diminished expression of DNAM-1 ligand CD155 on T cells (81, 99). Subsequent studies revealed that the expansion of CD56^bright^ NK cells after daclizumab was most likely due to the increased binding of IL-2 to the intermediate IL-2 receptor present on NK cells, which is not targeted by daclizumab (108, 109). Despite the beneficial effects, some MS patients developed autoimmune encephalitis after administration of daclizumab, possibly in part caused by the depletion of regulatory T cells, leading to withdrawal of the drug (110). Fingolimod, a sphingosine-1-phosphate receptor modulator, is a commonly used drug in current MS therapy. A recent study described an expansion of a distinct NK cell subset characterized by an aged phenotype (CD56^dim^CD16^++^KIR^+/-^NKG2A^-^CD94^-^CCR7^+/-^CX3CR1^+/-^NKG2C^-^NKG2D^+^NKp46^-^DNAM1^++^CD127^+^) upon fingolimod treatment (111). Although the authors could not demonstrate an association of any particular NK cell subset with treatment response in their pilot study, they do warn that the observed expansion of a more exhausted and possibly less functional NK cell subset might contribute to the observed adverse events such as infections and malignancies upon long-term fingolimod treatment (112). Cases such as daclizumab and fingolimod stress the importance of isolating the exact immune subsets propagating the established beneficial effects of the treatment and averting the unsolicited effects.

Because of such studies, NK cells are now at last being recognized as interesting targets for biomarker and therapy research in a myriad of autoimmune diseases (113). In MS, studies trying to correlate NK cell titers and functionality with prognosis and therapy outcome are underway (114–118), but more validation work is needed to establish a mechanistic basis for such correlations.

## Conclusion and Future Perspectives

From different lines of research, there is evidence that NK cells are likely to play a role in the pathogenesis of MS. NK cells are present in the CSF of MS patients and although their absolute numbers are relatively low, NK cells have strong cytotoxic activity to target stressed cells which include autologous activated immune cells. Genetic studies reveal polymorphisms in genes important for target cell recognition and for the cytotoxic activity of NK cells. NK cells are also important sources of cytokines that further regulate the immune system. Analysis of NK cells within successful MS treatments shows clear associations with changes in NK cell numbers and phenotype. Overall, the findings reviewed here are indicative of a more regulatory action of NK cells in the pathogenesis of MS, but further research is needed to validate this hypothesis. The importance of NK cells in EAE is also emerging in animal studies, but there is no consensus on their role.

The current state-of-the-art fails to uniformly capture and characterize the complete and intricate heterogeneity of the NK cell repertoire in MS, including both disease-promoting and disease-protective mechanisms, thereby hampering their successful application in the clinic. As current literature has often limited itself to descriptive studies of major NK cell subset frequencies with limited resolution and without many validated functional inferences, our knowledge of the exact mechanistic contribution of specific NK cell subsets to MS pathology remains superficial. The use of more unbiased approaches such as single-cell omics will allow for a more holistic understanding of the immune cell repertoire in MS, uncovering previously unidentified or underappreciated immune players.

As MS is a disease of the CNS, it will be crucial to assess whether specific NK cell subsets infiltrate the CNS by passing the blood-brain barrier from the periphery or whether a tissue-resident innate lymphoid subset, as have been identified in a myriad of other tissues (119), contributes to (the resolution of) inflammatory processes in local MS lesions. Finally, the discovery of clonal expansions of NK cells (120), reminiscent of those described for T and B cells (94, 121), is bridging the gap between the innate and adaptive immune system and merits investigation in the context of MS. In this regard, it is interesting to note that infection with Epstein-Barr virus (EBV) has recently been confirmed as an important contributor to MS risk (122, 123), making it intriguing to speculate that EBV infection also impacts the NK cell repertoire, possibly affecting MS immunopathology, as has been postulated for CMV (124).

In conclusion, there is a clear rationale and need for further investigation of NK cell subsets and their role in MS. As both MS genetics and immunology have shown that this role is clearly intertwined with the adaptive immune system, a fundamentally holistic approach is warranted, integrating both innate and adaptive immune players in MS. Such research will contribute to a better understanding of the complex disease pathogenesis and provide a platform for rational therapy design for this chronic autoimmune disorder.

## Author Contributions

JB reviewed the literature, wrote the first draft of the manuscript and produced the figures. AG and PM critically revised the manuscript. All authors contributed to the article and approved the submitted version.

## Funding

Research of the authors into NK cells in MS is specifically supported by the Belgian Charcot Foundation. Furthermore, research of AG is supported by the Research Fund KU Leuven (C24/16/045), the Research Foundation-Flanders (FWO GOA7219N), MS Liga Vlaanderen, the Queen Elisabeth Medical Foundation, the Progressive MS Alliance, and the Horizon2020 “MultipleMS” consortium (grant EU RIA 733161). Research of PM is supported by grants from the Research Foundation-Flanders (FWO, G0A3218N), by funding from the European Union’s Horizon 2020 research and innovation programme under grant agreement No. 779295 and by C1 grant (C16/17/010) of the KU Leuven.

## Conflict of Interest

AG has received consulting/travel fees and/or research funding from Novartis, Roche and Merck.

The remaining authors declare that the research was conducted in the absence of any commercial or financial relationships that could be construed as a potential conflict of interest.

## Publisher’s Note

All claims expressed in this article are solely those of the authors and do not necessarily represent those of their affiliated organizations, or those of the publisher, the editors and the reviewers. Any product that may be evaluated in this article, or claim that may be made by its manufacturer, is not guaranteed or endorsed by the publisher.
